# A Low-Cost, Portable Device for Detecting and Sorting Aflatoxin-Contaminated Maize Kernels

**DOI:** 10.3390/toxins15030197

**Published:** 2023-03-04

**Authors:** Haibo Yao, Fengle Zhu, Russell Kincaid, Zuzana Hruska, Kanniah Rajasekaran

**Affiliations:** 1 Geosystems Research Institute, Mississippi State University, Stennis Space Center, MS 39529, USA; 2 College of Mechanical Engineering, Zhejiang University of Technology, Hangzhou 310023, China; 3 Southern Regional Research Center, USDA-ARS, 1100 Allen Toussaint Blvd, New Orleans, LA 70124, USA

**Keywords:** aflatoxins, maize, portable, low-cost, NDFI detection, sorting, developing countries

## Abstract

Aflatoxin contamination of maize is a major food safety issue worldwide. The problem is of special significance in African countries because maize is a staple food. This manuscript describes a low-cost, portable, non-invasive device for detecting and sorting aflatoxin-contaminated maize kernels. We developed a prototype employing a modified, normalized difference fluorescence index (NDFI) detection method to identify potentially aflatoxin-contaminated maize kernels. Once identified, these contaminated kernels can be manually removed by the user. The device consists of a fluorescence excitation light source, a tablet for image acquisition, and detection/visualization software. Two experiments using maize kernels artificially infected with toxigenic *Aspergillus flavus* were implemented to evaluate the performance and efficiency of the device. The first experiment utilized highly contaminated kernels (71.18 ppb), while mildly contaminated kernels (1.22 ppb) were used for the second experiment. Evidently, the combined approach of detection and sorting was effective in reducing aflatoxin levels in maize kernels. With a maize rejection rate of 1.02% and 1.34% in the two experiments, aflatoxin reduction was achieved at 99.3% and 40.7%, respectively. This study demonstrated the potential of using this low-cost and non-invasive fluorescence detection technology, followed by manual sorting, to significantly reduce aflatoxin levels in maize samples. This technology would be beneficial to village farmers and consumers in developing countries by enabling safer foods that are free of potentially lethal levels of aflatoxins.

## 1. Introduction

Aflatoxin, one of the most potent naturally occurring toxins, is produced by toxigenic mold species, including *Aspergillus (A.) flavus* and *A. parasiticus* [1]. These mold species naturally inhabit soils and thrive well under high heat and humidity. Aflatoxin is carcinogenic and is linked to liver disease, cancer, childhood stunting, and high mortality in both humans and animals [2]. Thus, aflatoxin contamination in maize is a major food safety issue worldwide. Maize is a staple food source for many developing regions, such as sub-Saharan Africa (SSA) [3], where the prevailing climate favors fungal growth and aflatoxin contamination in maize. The occurrence of aflatoxin contamination is more prevalent in developing countries due to the lack of proper detection and preventive technology, management, and unfavorable climatic conditions.

As a consequence, the mean aflatoxin levels found in developing countries, such as those in Africa, have always exceeded the legally allowable limits set by the FDA (U.S. Food and Drug Administration) and the European Union (EU), as reported in an extensive study conducted in 2010–2018 [4]. In the United States, the FDA allows 20 µg/kg (ppb, parts per billion) total aflatoxin in food and up to 100 ppb aflatoxin in feed. The EU has more stringent regulations of 2 and 4 ppb aflatoxin B_1_ (AFB_1_) and total aflatoxin in food, respectively [5]. Many other countries have also introduced regulated levels for aflatoxins in food and feed as means of controlling the toxins, with aflatoxin levels in foods not to exceed 5 and 10 ppb for AB_1_ and total aflatoxin, respectively [6].

One major step in controlling aflatoxin contamination is detecting the toxin. However, current aflatoxin detection methods are cost-prohibitive for users in many developing countries. For example, the current United States Department of Agriculture (USDA)-approved methods for aflatoxin detection are expensive chemical-based analytical methods that effectively destroy the sample. These methods include chromatography methods, enzyme-linked immunoassays, and immuno-fluorometry-based rapid test kits (e.g., ROMER, Romer Labs, Inc, Newark, DE, USA or VICAM, Waters Corporation, Milford, MA, USA). The steps in the inspection process involve sampling, sample grinding, and extraction, followed by chemical analysis [7], making the entire labor-intensive detection process cost-prohibitive for farmers or traders on small village farms, particularly in the SSA region. Annual maize production varied greatly among small farms, averaging from 0.18 to 3.8 tons [8], depending on the region and farm size. Because of the lack of affordable and feasible methods to screen for aflatoxin contamination in these areas, quite often, aflatoxin contamination is ignored by small, family-based producers [9]. Therefore, the development of low-cost alternative approaches, including portable, rapid, and non-invasive technology for aflatoxin detection and removal in maize is sorely needed.

The research community has been continuing its effort to detect aflatoxin contamination rapidly and non-destructively in maize. One such attempt is the black light test [10], which involves the detection of bright greenish-yellow fluorescence (BGYF) emanating from contaminated kernels or a stream of coarsely ground maize meal under 365 nm ultraviolet (UV) light. This method provides rapid detection at a very low cost. A previous study showed that BGYF-based manual selection could be an effective way to screen aflatoxin-contaminated figs [11]. However, it is not a reliable test for aflatoxin-contaminated maize [12]. The reasons include the fact that fluorescence, in general, is emitted from the intermixed aflatoxin and the BGYF compound produced during fungal invasion. The black light only reveals the broad fluorescence (visible light range, 400–700 nm) response of the samples. Thus, the observation of BGYF is based on broad-spectrum fluorescence. The black light test results in high rates of false detection. For this reason, the black light test is only used as a presumptive test for initial screening and detection rather than quantitative or even qualitative aflatoxin determination. However, although the BGYF test is not suitable for screening corn samples, BGYF sorting could be used to remove infected kernels. Previous research has indicated that BGYF sorting could provide a way to reduce aflatoxin contamination [13] in maize.

One of the more recent developments in the detection of aflatoxin contamination is the use of spectral technology, including the use of fiber-optic spectrometry [14], fluorescence hyperspectral imaging [15], multispectral imaging [11,16], fluorescence spectroscopy [17], fluorescence spectroscopy and multispectral imaging [18], ultraviolet–visible–near-infrared spectra [19], and hyperspectral and microscopic imaging [20]. A fluorescence hyperspectral study [15] found that there exists a fluorescence shift toward longer wavelengths in the blue-green spectral region in fungal-infected maize kernels with high aflatoxin content. This discovery opened up the possibility of using two narrow wavelength bands for the detection of contaminated maize kernels under UV light [21]. The detection algorithm is based on fluorescence emission from the two narrow wavelength bands. Based on this detection method, Han et al. (2019) [22] developed a multispectral imaging system that incorporated two narrowly filtered bands (436 and 532 nm) for aflatoxin contamination detection in industrial maize samples. The system used two high-performance monochromatic cameras for high-speed image data acquisition. UV fluorescence has also been used for aflatoxin detection in other agricultural products. A recent study [18] using UV fluorescence spectra for maize kernel aflatoxin detection corroborated an earlier study [15] by finding a similar fluorescent shift associated with aflatoxin-contaminated kernels. The study reported a classification accuracy of 100% with principal component and linear discriminant analyses. Lunadei et al. (2013) [23] utilized UV fluorescence imaging to detect aflatoxins in naturally contaminated pistachio and cashew nuts. The research indicated that the optimal imaging bands were 480 and 520 nm for pistachio and 440 and 600 nm for cashews. In another study [11], multispectral UV fluorescence imaging was used to detection aflatoxin-contaminated figs. This study found that the spectral bands between 475 and 575 nm generated the highest correlations between BGYF and aflatoxin contamination. Fluorescence emission from samples can also be excited with laser light. Paghaleh et al. (2015) [24] used laser (308 nm)-induced UV fluorescence spectroscopy for aflatoxin detection in fungus-infected pistachio nuts.

Commercially, the Tomra company (Asker, Norway) has developed a high-speed fluorescence sorter. The sorter (OMRA Helius P640) was validated with peanut samples using a laser for fluorescence excitation and captured fluorescence emissions under certain, undisclosed wavelengths [25]. The sorter was built for industrial applications with a throughput of 2–4 tons per hour and is expensive. It is suitable for developed countries but not for developing countries.

With the advancement of modern technology, mobile computing devices such as smartphones and tablets are increasingly being used for the detection of biological contamination. One recent review [26] discussed smartphone-based imaging devices for the possible detection of areas contaminated with viruses and bacteria, as well as for food quality control. We determined that the above-mentioned narrow-fluorescence-wavelength-based approach could be further extended to the development of low-cost detection systems with mobile computing technology. Therefore, the objective of the present research was to develop portable, fluorescence-imaging-index-based technology for rapid and non-invasive aflatoxin detection in maize kernels with the objective of providing a simple, low-cost device for end users (farmers, traders, and consumers) in developing countries.

## 2. Results

Based on the calculated original aflatoxin level (ppb) of a given sample, a positive sample was defined as a sample equal to or above a 4 ppb aflatoxin concentration, the most stringent regulation for world export. A negative sample was defined as a sample containing less than 4 ppb aflatoxin. This is the ground-truth information established from chemical analysis regardless of the initial sample preparation. Based on this criterion, in experiment 1, the total number of negative samples was 12, and the number of positive samples was 87. One outlier in the contaminated sample group was removed because of an extreme aflatoxin value. During the detection process, an entire sample was labeled as positive if there were maize kernels identified as contaminated kernels and negative if no contaminated kernels were detected. Table 1 is the confusion matrix of the detection results from experiment 1. The results revealed the sensitivity (true positive rate) to be 100%, while the specificity (true negative rate) was 75%. The overall detection accuracy was 97%.

Among the 10 control samples, 9 samples had zero-aflatoxin readings, and they were correctly identified as negative samples. There was one control sample identified as positive, and it contained one positive kernel. The measured aflatoxin level of the positive kernel was 75 ppb. The original calculated aflatoxin level of the sample was 0.67 ppb. Chemically, the sample was regarded as a negative sample based on the 4 ppb criteria, as defined above. The result indicated that the “clean” maize kernels used for the control samples were not completely clean. Regardless of the detection error, the contaminated kernel in the negative sample was still identified and sorted out. For the purpose of aflatoxin reduction, the detection and sorting procedure works in practice.

**Figure 1:**
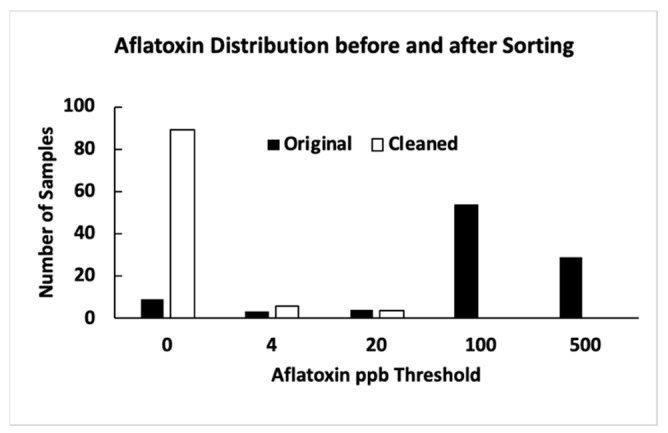
Aflatoxin distribution before and after sorting (experiment 1).

Among the 87 contaminated samples, all were identified as positive. There were two samples with aflatoxin levels of less than 4 ppb (0.03 and 0.31 ppb). Similar to the above-mentioned control samples, these two contaminated samples were chemically regarded as negative. During detection, they were identified as positive since each had a positive kernel (7 and 60 ppb). Additionally, just as above, the results led to a sample detection error but, from a practical perspective, helped reduce aflatoxin levels through the detection and sorting procedure. The sorting process divided each contaminated sample into two groups: positive detection and negative detection. The “positive detection” group had aflatoxin levels ranging from 7 to 12,500 ppb. The “negative detection” group ranged from 0 to 15 ppb. In the negative detection group, 80 samples had 0 ppb. Among the other nine samples in the negative detection group, five contained less than 4 ppb aflatoxin (0.43, 1.3, 1.8, 2.4, and 3.85 ppb), and four exceeded the 4 ppb level (4.05, 6.5, 13.5, and 15 ppb). As discussed earlier, the reason for the low aflatoxin levels in some sorted negative samples could be because the original “clean” kernels were not completely clean. Additionally, since the detection was based on only one side of a maize kernel, if partial fluorescence associated with fungal infection was on another side of the kernel, it may have escaped the detection procedure implemented in this research. Figure 1 is an aflatoxin distribution histogram for experiment 1. The distribution illustrates the efficacy of removing aflatoxin-contaminated kernels from the samples. The number of samples containing >4 ppb was reduced from 87 to 4.

While experiment 1 worked as a baseline experiment to test the performance of the prototype device using highly contaminated kernels, it did not reflect a real-world situation, where aflatoxin contamination is generally skewed to a few heavily contaminated kernels. Silk inoculation in experiment 2 induced a more realistic fungal invasion. Table 2 shows the detection accuracy results of experiment 2. There were 5 positive samples and 94 negative samples, confirmed by chemical analysis (with 1 outlier removed due to a sorting error). Sensitivity (true-positive rate) was found to be 100%, while specificity (true-negative rate) was 22.3% (21/94). The false-positive rate was 77.7% (73/94), and the overall detection accuracy was 26.3%. These results suggest that due to the high false-positive rate, the approach is not suitable for aflatoxin screening. Similar outcomes were reported in a previous study [16] using multispectral data for aflatoxin sorting in maize obtained from local markets in Kenya. It was mentioned that only half of the kernels exhibiting fluorescence had aflatoxin readings > 10 ppb. The production of high-aflatoxin-containing kernels is skewed to a small portion of maize. In a real-world scenario, fungal infection may not produce aflatoxins if the conditions (temperature, drought stress, etc.) are not favorable, indicating that infected kernels might not contain aflatoxin. Since the fluorescence-based method detects fungal infection, it could lead to high levels of false positives if aflatoxin is not produced.

**Figure 2:**
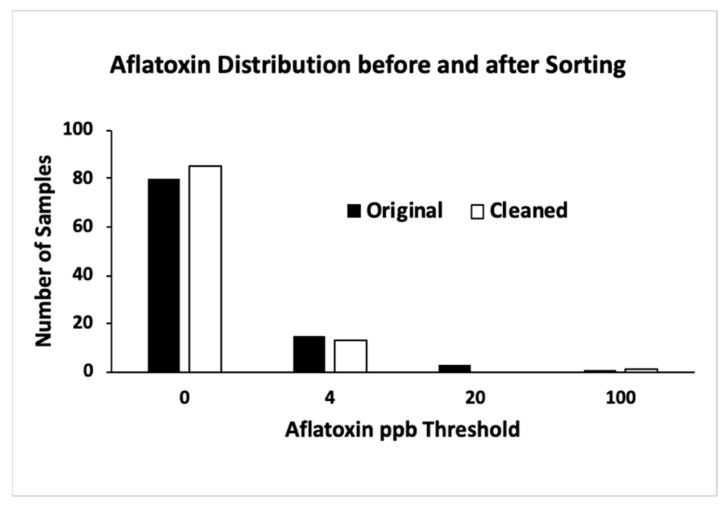
Aflatoxin distribution before and after sorting (experiment 2).

The five positive samples had original aflatoxin levels of 4.0, 5.1, 15.3, 16.0, and 64.6 ppb. After detection and sorting, aflatoxin levels in the negative group of the five samples were reduced to 0, 0, 0, 0.9, and 55 ppb, respectively. Conversely, the positive samples were 1400, 95, 1000, 1400, and 1500, respectively. The sample with an original level of 64.6 ppb had the most contamination, and only a portion of the contamination was removed. Among the 94 negative samples, 80 had zero-aflatoxin readings. The other 14 contained less than 4 ppb aflatoxin each. Figure 2 is an aflatoxin distribution histogram from experiment 2. The number of samples containing >4 ppb aflatoxin level was reduced from five to one after the detection and sorting operation.

**Figure 3:**
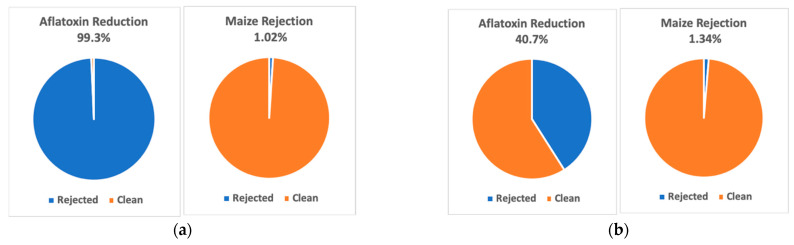
Maize rejection and aflatoxin reduction ratios for experiments 1 and 2. (**a**) Experiment 1, (**b**) Experiment 2.

The other performance metric evaluated was aflatoxin reduction. In the 99 samples in experiment 1, the mean sample aflatoxin concentration was 71.18 ppb. After the detection and sorting process, the mean aflatoxin reading for the 99 cleaned (negative detection) samples was 0.49 ppb, while the mean aflatoxin reading for the contaminated (positive detection) samples was 6921 ppb. Collectively, the sorting process removed 54.03 g of contaminated kernels from a total of 5290 g of maize mass. This amounts to a rejection ratio of 1.02%, from which an aflatoxin reduction efficiency of 99.3% was achieved (Figure 3a) for kernels with severe infection from side-needle inoculation.

As for the 99 samples in experiment 2, the original mean sample aflatoxin level was 1.22 ppb. After the detection and sorting process, the mean aflatoxin level for the 99 cleaned (negative detection) samples was 0.72 ppb. The mean aflatoxin level for the five removed positive-detection samples was 983 ppb. The mean aflatoxin level for all of the removed samples was 37.54 ppb. Collectively, the sorting process removed 62.41 g of contaminated kernels from a total of 4647.41 g of maize mass. This amounts to a maize rejection ratio of 1.34%, from which a mean aflatoxin reduction ratio of 40.7% was achieved (Figure 3b). Although the results of experiment 2 were not as impressive as those of experiment 1, they were nonetheless significant for kernels with mild infection from silk inoculation.

Sorting is an effective way of reducing aflatoxin levels in maize. Affordability is crucial in implementing sorting strategies in developing countries. Several studies implemented low-cost technology for maize aflatoxin sorting and reduction. One study [16] used a multispectral sorting approach with a laboratory-scale sorter [27]. The rejection rate was 0–1% with kernels previously testing aflatoxin-negative and 0–25% with kernels testing positive. The aflatoxin reduction rate was 83%. The advantage of the device was its theoretical throughput of 25 kg/h, which is suitable for small-scale milling operations in developing countries. Another low-cost approach is kernel density sorting [13,28]. It was reported that the density-sorting-based DropSort device [12] may be useful in reducing aflatoxin levels but was not effective in reducing aflatoxin levels below 20 ppb in maize. Conversely, a different density sorting study [29] using a screen and a gravity table was able to reduce aflatoxin levels below 20 ppb. Hence, sorting based on kernel physical properties could be influenced by many factors, including differences in the kernel genotype, growth conditions, infection and contamination conditions, and sorting methods. As a holistic approach, a low-cost sorting strategy could have a multiple-stage approach utilizing the physical properties of kernels in addition to their fluorescence features. 

## 3. Conclusions

While large-scale industrial sorters are available in developed countries, simple and affordable sorting devices are preferred for users in small village farms in developing countries. They will be valuable for screening limited quantities of corn kernels just before meal preparation. The sorting of fungal-infected and aflatoxin-contaminated maize kernels is an effective way to reduce aflatoxin contamination in a bulk sample. The removal of these compromised kernels could further reduce the contamination problem caused by inadequate storage conditions, which is not uncommon in developing countries. The goal of the present research was to develop low-cost aflatoxin detection and sorting technology to remove aflatoxin-contaminated maize kernels. The developed system uses the fluorescence band ratio to identify maize kernels infected by fungi that cause aflatoxin contamination. The research successfully developed a prototype device including hardware with integrated software. The detection algorithm was incorporated into an in-house-developed software application on a tablet device. Experimental results showed that the prototype was effective in reducing contamination levels in highly contaminated maize kernels (99.3%). A moderate aflatoxin reduction (40.7%) was achieved with maize samples where the contamination was minor and skewed, reflecting real-life field-induced conditions. In both cases, the maize rejection ratio was approximately one percent of the sample lot. This research demonstrated that the simple fluorescence-band-ratio-based approach is effective in aflatoxin sorting and reduction. In addition, the research could be expanded to real-world maize samples obtained from local African markets to test the efficacy of the detection device. Furthermore, it is expected that, in the future, the detection software developed in the current study will be directed toward other commodities and transferred to alternate mobile devices, including smartphones. With the widespread use of smartphones in developing countries, the cost of using low-cost aflatoxin detection and reduction technology could be further reduced. Finally, this mobile technology also provides the potential of establishing the large-scale monitoring of aflatoxin outbreaks by uploading and sharing the detection results from a public network of devices.

## 4. Materials and Methods

### 4.1. Instrumentation

**Figure 4:**
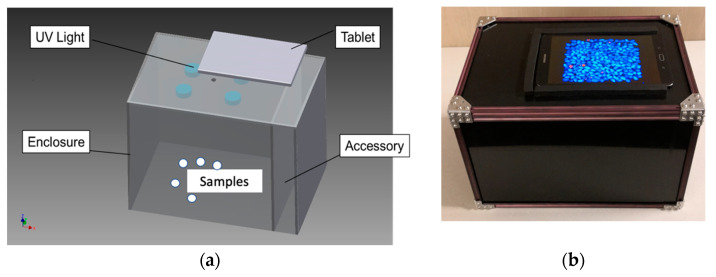
The portable detection device. (**a**) Prototype detection device component diagram, (**b**) Finished device.

In this research, a portable imaging detection device was designed, built, and tested for rapid aflatoxin contamination detection and removal. This section focuses on the specifications and design of the portable device. For maize sample imaging, the image size was set equivalent to the size of A4 paper, or 215 mm × 325 mm, at an imaging height of 210 mm. The final sample tray size, with a capacity to hold 50 g of kernels, was defined as 137 × 137 mm in size based on the actual camera’s field of view. Based on the imaging requirement, an imaging enclosure was designed and built so that all imaging operations were kept in a dark environment to restrict the influence of ambient light. Another reason for a closed imaging environment was safety. Because the detection device uses UV illumination as the light source, which is harmful to exposed eyes and skin, the enclosure will keep the UV light away from operators/bystanders. The enclosure has dimensions of 350, 240, and 230 mm (Length × Height × Width). Figure 4a is a component diagram of the imaging device. Other major components included are a UV-LED (light-emitting diode) array, a pair of emission and excitation filters, and a tablet for real-time imaging and detection. The UV-LED array consists of 4 UV-LED chips, each with 20 × 1w UV-LEDs. Thus, the total LED power output of the imaging device is 80 w. The UV-LED chips were custom-made per the requirement of the research (Figure S1a). To standardize the spectral output of the UV-LED array, a 365 nm UV excitation filter is placed in front of the UV-LED chips to filter the spectral curve (Figure S1b). The UV-LED array is attached to the inner ceiling of the enclosure (Figure 4a). Since the UV-LEDs generate significant heat during their operation, an aluminum mounting bracket was used to also serve as a heat sink. The UV-LED array is powered by a 120 V AC/DC adaptor (or solar power in Figure S2a). The adaptor is mounted in the accessory compartment, separate from the main imaging area. In addition, an emission filter with a cut-off wavelength of 400 nm UV was mounted in front of the tablet camera. The role of the emission filter is to block any UV light below 400 nm from entering the camera. This ensures that the camera only records fluorescence signals excited by the UV light and does not include stray UV light from the light source. To add portability, a solar panel and a portable battery could be included to assist with the off-grid use of the detection device (Figure S2a). It was estimated that the total cost of the device would be approximately USD 200 if produced in large quantities. For comparison, an earlier investigation [15] working on a low-cost spectral-based sorting device used a circuitry board priced under USD 100.

Figure 4b illustrates the completed prototype device and operations. The sample tray can hold approximately 50 g of maize samples (Figure S2a). The black background of the sample tray is convenient for masking out the background from the maize kernels in the foreground during image processing. A Samsung Tab S2 8” tablet was used for image acquisition and processing. The rear camera has an 8.0 MP (megapixel, 3264 × 2448) image size. Another major component of the portable device is an Android application named AFSort. The application was developed in Java^®^ in the Android Studio. The main functions of AFSort are to acquire fluorescence images of maize samples, process images for contamination detection, display the detection result for contamination assessment, and assist with manual removal. The details are presented in the next section.

### 4.2. Method and Software Development

In this research, aflatoxin contamination detection in fluorescence images of maize samples was implemented based on a modified normalized difference fluorescence index (NDFI) developed previously [21]. In the previous study, the NDFI was calculated using two narrow hyperspectral bands, 437 and 537 nm. Because images acquired by a tablet are broadband red (R), green (G), and blue (B) images, narrow-band spectral indices cannot be directly calculated. Since the two NDFI hyperspectral bands are located in the blue (437 nm) and green (537 nm) band range in RGB images, the detection method was modified to calculate the NDFI using the blue and green bands, as illustrated in Equation 1.

(1) $$NDFI=\frac{B-G}{B+G}$$

where *B* and *G* are blue and green band fluorescence intensities from the fluorescence images captured by the tablet. The intensity is the digital number (0–255) for each band recorded by an 8-bit camera. 

**Figure 5:**
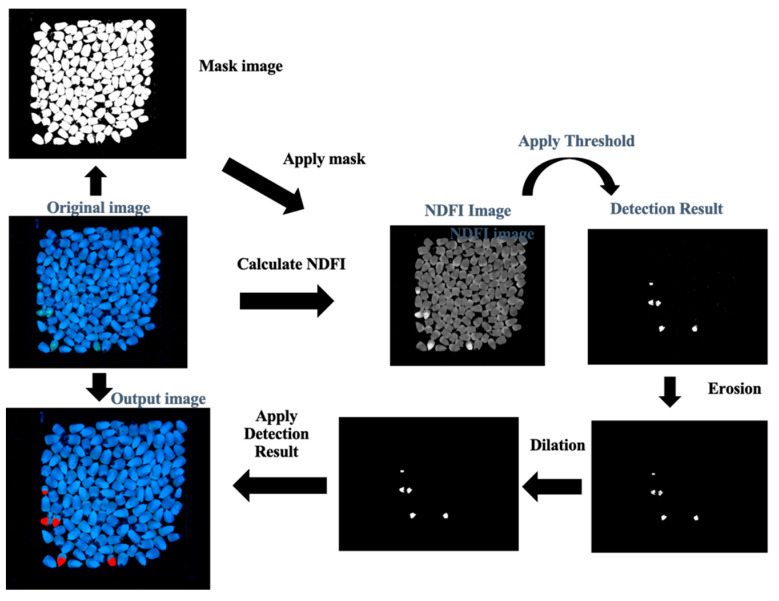
Flowchart of the image analysis steps for kernel aflatoxin contamination detection.

Figure 5 describes the image-processing steps that implement the detection algorithm in Equation 1. In this process, the operator first loads approximately 50 g of maize kernels and spreads them into a single layer on a sample tray. The tray is then placed in the imaging enclosure, and the UV light is turned on. In the AFSort application, the operator is prompted to take a fluorescence image of the maize sample. Once the sample image is confirmed by the operator, AFSort goes through a sequence of image-processing steps shown in Figure 4 for contamination detection. Briefly, a binary image of the sample image is first generated and used to create a mask image to separate the maize kernel pixels from the background pixels. The blue and green bands in the image are extracted to calculate the NDFI. A detection threshold is applied to the NDFI image to generate the initial classification result. Two post-classification image-processing procedures, erosion and dilution, are applied to remove the small and scattered noisy points to produce the final classification result. The final result is superimposed back to the original fluorescence image as the final classification image. The original and final images are stored in the tablet and also displayed to the operator for a subsequent sorting operation.

### 4.3. Experiments

Based on the above description, the detection device can be used in two approaches. One approach is to apply the detection as a screening tool for potential contamination detection of the entire sample. The other approach is to apply the detection to assist in the sorting and removal of potentially contaminated maize kernels. Thus, experiments implemented to test the portable prototype detection device were defined as screening (detection) and sorting (detection and removal) processes. These experiments were designed to detect and sort out contaminated maize kernels from bulk samples. The maize sample was first imaged to identify contaminated kernels. The identified kernels were then sorted out manually from the rest of the sample. Since the detection algorithm was pixel-based, partially identified maize kernels were all regarded as contaminated kernels and removed. After each imaging and sorting, the maize sample was separated into two sub-groups, positive and negative, indicating contaminated and cleaned. The two sub-group samples were then chemically analyzed to determine their actual aflatoxin concentrations.

**Figure 6:**
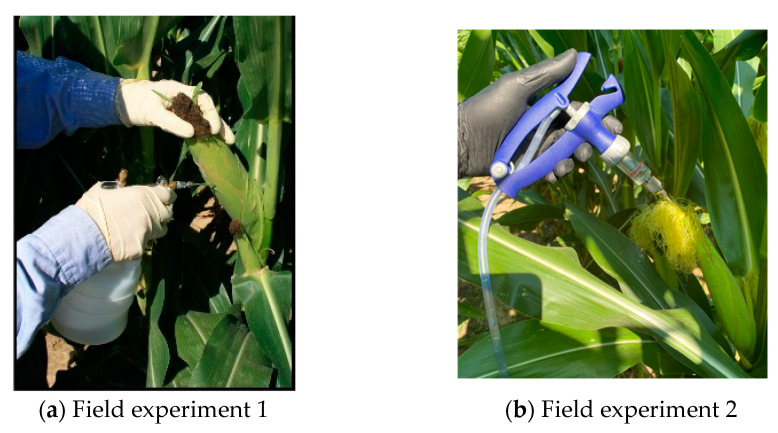
Inoculation method: (**a**) side-needle inoculation and (**b**) silk inoculation.

There were two experiments implemented using the prototype device. Both experiments used field-inoculated maize kernels. Field inoculation was conducted in the early dough stage of maize ear development. The purpose of field inoculation was to artificially inoculate maize ears with *Aspergillus flavus* (*AF13*) spores to produce aflatoxin-contaminated maize kernels. *AF13* is an aflatoxin-producing strain of *A. flavus*. The fungus was acquired from USDA-ARS, Southern Regional Research Center in New Orleans, Louisiana, and the inoculum was prepared based on [21]. Field experiment 1 used a side-needle inoculation approach (Figure 6a) and field experiment 2 used a silk inoculation approach (Figure 6b). For side-needle inoculation, 3.0 mL of the inoculum was injected into the side of each ear with a 12-gauge stainless steel needle through the husk. Since the side-needle approach caused more physical damage to maize ears (mimicking insect damage), it resulted in more severely contaminated maize kernels. The alternate inoculation method simulated natural field inoculation via silk inoculation (Figure 6b), which produced mildly contaminated kernels. During silk inoculation, the inoculum was injected into the exposed silk near the natural opening in the husk at the top of each ear. Field experiment 1 (with maize var. Syngenta N78S-311) was conducted in Stoneville, MS, in 2015. Field experiment 2 (with var. Pioneer P1184HR) was conducted in 2016, also in Stoneville, MS.

After inoculation, maize ears were allowed to mature in their natural state until harvest. Both control and inoculated ears were harvested two months post-inoculation. At the time of harvest, the maize plants were senescent, and the kernels were naturally dried to below 15% moisture content. If the moisture level in maize ears was too high, they were oven-dried immediately after harvest to below 15% moisture. The dried ears were then hand-shelled. For experiment 1, whole, undamaged kernels near the injection site on the maize ears were selected as potentially contaminated maize. Control kernels were obtained from un-inoculated ears harvested from the same field. For experiment 2, whole, undamaged kernels were selected from areas of the ear that exhibited visible mold growth on the surface of the kernels.

In this study, both experiments were completed within one year of maize harvest. The purpose of experiment 1 was to test the function of the prototype with baseline pre-mixed contaminated maize samples. It also represented instances of heavily contaminated maize. There were 100 samples used in the experiment, with 10 control and 90 contaminated samples. To prepare the baseline samples, both “clean” and “contaminated” maize kernels were initially obtained from shelled maize in field experiment 1. The “clean” kernels were from unaltered ears of field-grown corn. The “contaminated” maize kernels were whole, intact kernels from around the needle injection points of field-inoculated maize ears that exhibited BGYF. To prepare each contaminated sample, approximately 50 g of “clean” kernels was mixed with a random number of 1–10 “contaminated” kernels. Each control sample contained approximately 50 g of “clean” kernels. After sample preparation, each sample was examined and sorted with the prototype device using the detection and sorting procedure described above to split the sample into two groups, negative detection and positive detection, followed by aflatoxin analysis to evaluate the detection results. 

The shelled kernels obtained from silk inoculation were used in experiment 2. Each of the 100 samples was randomly prepared from the shelled maize. Thus, experiment 2 represented a more realistic sorting requirement for aflatoxin contamination. Additionally, the contamination levels in experiment 2 samples were expected to be much lower compared to those in experiment 1. The samples went through the same detection and sorting procedure as before; i.e., each sample was sorted into a negative or positive group. Chemical analysis for aflatoxin determination was implemented on the sorted maize (negative detection) as well as the contaminated kernels that were sorted out (positive detection). 

For the evaluation of results, aliquots of each sample, both negative detection and positive detection, were analyzed to determine the actual aflatoxin concentrations. The analysis was performed using the immunoaffinity-based AflaTest from VICAM (Waters Corporation, Milford, MA, USA). The AflaTest method uses an immunoaffinity column to isolate aflatoxins B1, B2, G1, and G2 (total aflatoxin), for fluorometric detection and quantification at the parts-per-billion (ppb) level. The 50 g samples were processed following the protocol for corn in the AflaTest WB SR instruction manual (Waters Corporation, Milford, MA, USA). Briefly, each 50 g sample was blended with 5 g of sodium chloride and 100 milliliters (mL) of methanol–distilled water (80:20, *v*:*v*) for 1 min. Extracts were filtered through a fluted filter, diluted (1:4) with distilled water, followed by filtration through a glass, microfiber filter, and passed through the affinity columns. The columns were washed twice with distilled water and eluted with pure methanol into a test tube. The eluents were mixed with a developer, and the contents of each tube were measured with a calibrated Series 4 EX fluorometer from VICAM. The protocol was modified for small samples by adjusting the extraction liquid based on the weight of each sample, adding 2 mL for each 0.1 g. The details of the protocol were reported previously [30].

### 4.4. Analysis

Two metrics, detection accuracy and aflatoxin reduction, were used in this study. Detection accuracy (Supplementary Materials Equation (S1)) was used to assess the detection outcome of each sample with the prototype device. The aflatoxin reduction ratio (Supplementary Materials Equation (S2)) was used to evaluate the overall performance of the aflatoxin removal procedure defined above, including aflatoxin detection and sorting with the prototype device. The confusion matrix was also calculated to show the detection accuracy distribution in different sample groups. In addition, sensitivity (true-positive rate) and specificity (true-negative rate) were also calculated.

As described previously, in the experiment, each sample was separated into negative and positive groups after sorting. Based on the measured aflatoxin ppb levels from the negative and positive groups, the original ppb for each sample was calculated using Supplementary Materials Equation (S3). In addition, the “Average Original lot ppb” was calculated using all samples in an experiment. The “Average Clean lot ppb” was also calculated for the sorted clean samples. Lastly, the sample rejection ratio (Supplementary Materials Equation (S4)) was calculated to assess the percentage of removed kernels.

## Figures and Tables

**Table 1:** Detection results of experiment 1.

		Detection		
		Positive	Negative	
Actual (4 ppb as threshold)	Positive	87	0	87
	Negative	3	9	12
		90	9	
		Detection accuracy		97%

**Table 2:** Detection results of experiment 2.

		Detection		
		Positive	Negative	
Actual (4 ppb as threshold)	Positive	5	0	5
	Negative	73	21	94
		78	21	
		Detection accuracy		26.3%

## Data Availability

Not applicable.

## Supplementary Materials

The following supporting information can be downloaded at https://www.mdpi.com/article/10.3390/toxins15030197/s1. Figure S1: UV-LED properties; Figure S2: The prototype portable aflatoxin contamination detection device; Equation (S1): Detection accuracy; Equation (S2): Aflatoxin reduction ratio; Equation (S3): Original sample ppb; Equation (S4): Rejection ratio.
