# Supplementary material for: A Low-Cost, Portable Device for Detecting and Sorting Aflatoxin-Contaminated Maize Kernels
Source: Toxins (Basel). 2023 Mar 4;15(3):197. doi: 10.3390/toxins15030197 (PMC10058786; doi:10.3390/toxins15030197)
Supplement: Supplementary file 1 [file toxins-15-00197-s001.zip › toxins-2188380-supplementary.pdf]

toxins

MDPI

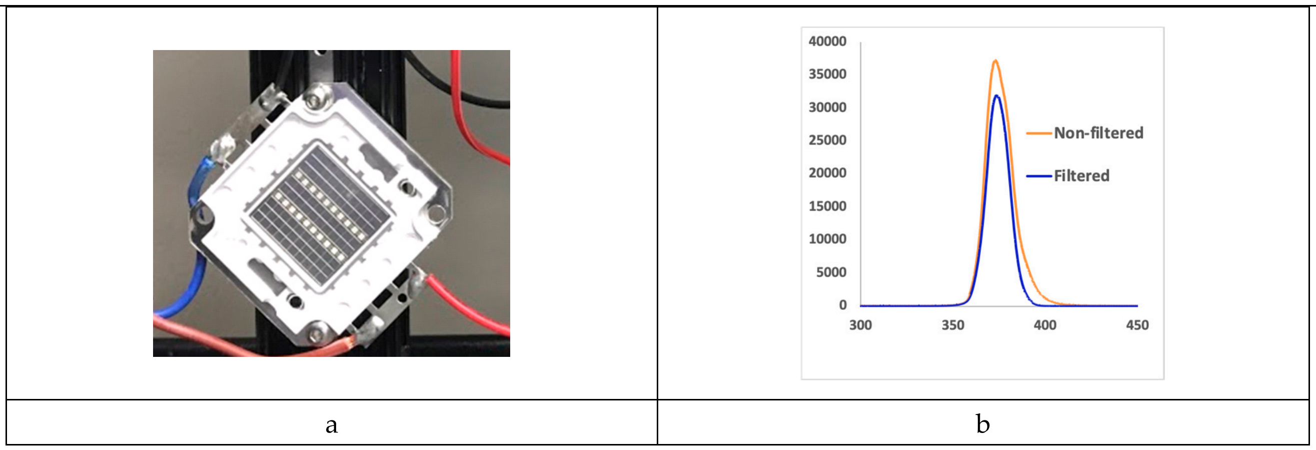

a b

Figure S1. UV-LED properties. One 20 W UV-LED chip is embedded with 20 × 1 W UV-LEDs. The UV-LED light was filtered by 365 nm excitation filter. The spectral data was collected with an Avantes (Apeldoorn, Netherlands) UV-Visible spectrometer. **a** .UV-LED chip with 20 1-w LEDs, **b**. UV-LED Spectra (in nm).

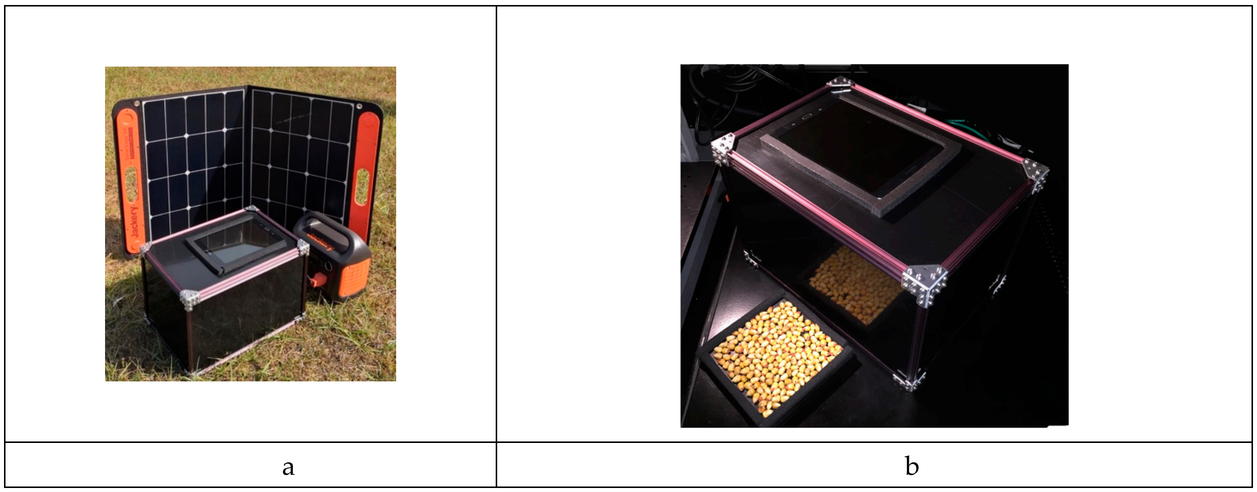

a b

Figure S2. The prototype portable aflatoxin contamination detection device employs a tablet for fluorescence imaging of maize samples. **a**. Device with solar power, **b**. Prototype detection device with sample tray.

Equations used in Analysis:

Detection accuracy is defined in equation S1:

$$Detection\ Accuracy = \frac{Number\ of\ correctly\ detected\ samples}{Total\ number\ of\ samples} \times 100\% \quad \text{(S1)}$$

Aflatoxin reduction ratio was calculated for each experiment. In this case, each experiment was regarded as a maize lot, each weighing approximately 5 kg. Thus, the reduction ratio is described in equation S2:

$$Aflatoxin\ reduction\ Ratio = \frac{Average\ Original\ \mathrm{lot}\ ppb - Average\ Clean\ lot\ ppb}{Average\ Original\ \mathrm{lot}\ ppb} \times 100\% \quad \text{(S2)}$$

$$Orig.Sample\ ppb = \frac{Weight_{positive} * ppb_{postive} + Weight_{negative} * ppb_{negative}}{Total\ sample\ weight} \quad \text{(S3)}$$

$$Rejection\ ratio = \frac{Total\ rejected\ Weight}{Total\ lot\ weight} \times 100\% \quad \text{(S4)}$$

Where the “Total rejected weight” was the pooled weight of all rejected samples from one experiment.
